# Supplementary material for: Modeling glioblastoma heterogeneity as a dynamic network of cell states
Source: Mol Syst Biol. 2021 Sep 16;17(9):e10105. doi: 10.15252/msb.202010105 (PMC8444284; doi:10.15252/msb.202010105)
Supplement: Supplementary file 6 — Source Data for Figure 5 [file MSB-17-e10105-s004.zip › Figure5A_sourcedata/GSEA_3017/hallmarks_stateD.GseaPreranked.1621934542601/HALLMARK_TNFA_SIGNALING_VIA_NFKB.html]

Details for gene set HALLMARK\_TNFA\_SIGNALING\_VIA\_NFKB[GSEA]

|  || Dataset | state23017 |
| Phenotype | NoPhenotypeAvailable |
| Upregulated in class | na\_neg |
| GeneSet | HALLMARK\_TNFA\_SIGNALING\_VIA\_NFKB |
| Enrichment Score (ES) | -0.36128545 |
| Normalized Enrichment Score (NES) | -1.5529587 |
| Nominal p-value | 0.052927926 |
| FDR q-value | 0.16356847 |
| FWER p-Value | 0.761 |
Table: GSEA Results Summary

  

Fig 1: Enrichment plot: HALLMARK\_TNFA\_SIGNALING\_VIA\_NFKB      
 Profile of the Running ES Score & Positions of GeneSet Members on the Rank Ordered List

  

| PROBE | GENE SYMBOL | GENE\_TITLE | RANK IN GENE LIST | RANK METRIC SCORE | RUNNING ES | CORE ENRICHMENT || 1 | FJX1 |  |  | 12 | 0.514 | 0.0280 | No |
| 2 | BTG1 |  |  | 50 | 0.350 | 0.0248 | No |
| 3 | TGIF1 |  |  | 55 | 0.343 | 0.0465 | No |
| 4 | PHLDA1 |  |  | 59 | 0.336 | 0.0686 | No |
| 5 | MYC |  |  | 77 | 0.313 | 0.0782 | No |
| 6 | MARCKS |  |  | 96 | 0.295 | 0.0856 | No |
| 7 | IER2 |  |  | 101 | 0.290 | 0.1035 | No |
| 8 | MCL1 |  |  | 115 | 0.279 | 0.1137 | No |
| 9 | PPP1R15A |  |  | 149 | 0.259 | 0.1069 | No |
| 10 | TANK |  |  | 238 | -0.258 | 0.0575 | No |
| 11 | NAMPT |  |  | 450 | -0.279 | -0.0855 | No |
| 12 | ABCA1 |  |  | 563 | -0.293 | -0.1510 | No |
| 13 | BTG3 |  |  | 581 | -0.296 | -0.1427 | No |
| 14 | CSF1 |  |  | 614 | -0.303 | -0.1454 | No |
| 15 | TUBB2A |  |  | 677 | -0.315 | -0.1706 | No |
| 16 | TIPARP |  |  | 841 | -0.355 | -0.2710 | No |
| 17 | PMEPA1 |  |  | 868 | -0.363 | -0.2648 | No |
| 18 | CFLAR |  |  | 873 | -0.364 | -0.2415 | No |
| 19 | PDLIM5 |  |  | 941 | -0.390 | -0.2650 | No |
| 20 | PLK2 |  |  | 1047 | -0.444 | -0.3141 | No |
| 21 | SAT1 |  |  | 1109 | -0.473 | -0.3270 | Yes |
| 22 | ATP2B1 |  |  | 1129 | -0.495 | -0.3058 | Yes |
| 23 | CEBPD |  |  | 1158 | -0.520 | -0.2897 | Yes |
| 24 | SERPINE1 |  |  | 1218 | -0.617 | -0.2905 | Yes |
| 25 | F3 |  |  | 1241 | -0.673 | -0.2588 | Yes |
| 26 | PTX3 |  |  | 1242 | -0.679 | -0.2095 | Yes |
| 27 | CD44 |  |  | 1270 | -0.762 | -0.1751 | Yes |
| 28 | CDKN1A |  |  | 1306 | -1.046 | -0.1264 | Yes |
| 29 | CCL2 |  |  | 1317 | -1.880 | 0.0023 | Yes |
Table: GSEA details [plain text format]

  

Fig 2: HALLMARK\_TNFA\_SIGNALING\_VIA\_NFKB: Random ES distribution      
 Gene set null distribution of ES for **HALLMARK\_TNFA\_SIGNALING\_VIA\_NFKB**
